# Nanoengineered, cell-derived extracellular matrix influences ECM-related gene expression of mesenchymal stem cells

**DOI:** 10.1186/s40824-018-0141-y

**Published:** 2018-10-05

**Authors:** Hatice O. Ozguldez, Junghwa Cha, Yoonmi Hong, Ilkyoo Koh, Pilnam Kim

**Affiliations:** Department of Bio and Brain Engineering, KAIST, Daejeon, 34141 South Korea

**Keywords:** Nano-engineered ECM, Decellularization, Mesenchymal stem cells

## Abstract

**Background:**

Human mesenchymal stem cells (hMSCs) are, due to their pluripotency, useful sources of cells for stem cell therapy and tissue regeneration. The phenotypes of hMSCs are strongly influenced by their microenvironment, in particular the extracellular matrix (ECM), the composition and structure of which are important in regulating stem cell fate. In reciprocal manner, the properties of ECM are remodeled by the hMSCs, but the mechanism involved in ECM remodeling by hMSCs under topographical stimulus is unclear. In this study, we therefore examined the effect of nanotopography on the expression of ECM proteins by hMSCs by analyzing the quantity and structure of the ECM on a nanogrooved surface.

**Methods:**

To develop the nanoengineered, hMSC-derived ECM, we fabricated the nanogrooves on a coverglass using a UV-curable polyurethane acrylate (PUA). Then, hMSCs were cultivated on the nanogrooves, and the cells at the full confluency were decellularized. To analyze the effect of nanotopography on the hMSCs, the hMSCs were re-seeded on the nanoengineered, hMSC-derived ECM.

**Results:**

hMSCs cultured within the nano-engineered hMSC-derived ECM sheet showed a different pattern of expression of ECM proteins from those cultured on ECM-free, nanogrooved surface. Moreover, hMSCs on the nano-engineered ECM sheet had a shorter vinculin length and were less well-aligned than those on the other surface. In addition, the expression pattern of ECM-related genes by hMSCs on the nanoengineered ECM sheet was altered. Interestingly, the expression of genes for osteogenesis-related ECM proteins was downregulated, while that of genes for chondrogenesis-related ECM proteins was upregulated, on the nanoengineered ECM sheet.

**Conclusions:**

The nanoengineered ECM influenced the phenotypic features of hMSCs, and that hMSCs can remodel their ECM microenvironment in the presence of a nanostructured ECM to guide differentiation into a specific lineage.

**Electronic supplementary material:**

The online version of this article (10.1186/s40824-018-0141-y) contains supplementary material, which is available to authorized users.

## Background

Human mesenchymal stem cells (hMSCs) are multipotent adult stem cells that can differentiate into adipocytes, osteoblasts, or chondrocytes [1–4]. hMSCs can be isolated from various tissues and their use raises no ethical issues. Therefore, hMSCs are useful sources for stem cell therapy and tissue regeneration; for the latter application, hMSCs are believed to differentiate and replace damaged cells.

In addition to their differentiation potential, environment-remodeling activity of MSCs may also affect the success of hMSC-based therapies. For example, hMSCs have immunomodulatory and trophic properties [5–7]. hMSCs secrete anti-inflammatory cytokines, induce cell proliferation and angiogenesis, inhibit apoptosis, and stimulate adjacent cells to induce tissue regeneration [5]. hMSCs may also remodel and optimize the extracellular matrix (ECM) that are an important component of the cellular niche in a tissue, supplying critical biochemical and physical signals to initiate and sustain cellular functions [8]. Indeed, the cell-ECM interaction is reciprocal; cells continually remodel the ECM in their microenvironment, and this remodeling in turn influences cell behavior and regulates ECM-mediated signaling [9–11].

It has been evidenced that the behavior and differentiation of hMSCs are regulated by ECM organization and composition [12–14]. In particular, the physical properties of the ECM, such as its rigidity, topography and porosity, modulate the maintenance, proliferation, self-renewal, and differentiation of hMSCs [15]. Indeed, the differentiation capacity of hMSCs on engineered matrices, such as decellularized ECM [15–17] or nanostructured surfaces [18–23] has been reported. For example, an hMSC-derived decellularized ECM was used to support the in vitro growth of newly seeded cells [24–27] and differentiation of stem cells [28–31]. Chen’s group reported that differentiation of hMSCs into specific lineages resulted in dynamic changes in the composition of ECM proteins and that sequentially promoted further differentiation of hMSCs [28–30].

Nanotopography that mimics the physical microenvironment can provide geometrical cues that influence cell fate [19–22, 31, 32]. Due to a reciprocal interaction between cell-ECM, the alteration within the cell in response to nanotopography can affect the microenvironment via nanotopography-mediated ECM remodeling. However, the mechanism involved in ECM remodeling by hMSCs under topographical stimulus is unclear.

For this purpose, we examined the effect of nanotopography on the expression of ECM proteins by hMSCs by analyzing the quantity and structure of the ECM on a nanogrooved surface. The decellularization of ECM deposited by hMSC cultured on the nanogrooved surface was performed through the chemical treatments. We verified the architecture of the hMSC-derived ECM sheet through immunofluorescence staining of the major ECM proteins. The decellularized ECM and nanotopography have been widely used to mimic biologically relevant characteristics of the ECM however, they have never been combined before to analyze dual effect on differentiation of hMSCs nor ECM remodeling by hMSCs. ECM produced by hMSCs on the nanotopography provided structured ECM proteins which mimics both biochemical and physical microenvironment in vivo. After successfully obtained decellularized ECM on nanotoporaphy, we elucidated the effect of hMSC-derived ECM on the reseeded hMSCs. We analyzed the morphology, focal adhesion, and ECM-related gene expression of the reseeded hMSCs. This model allowed us to analyze ECM remodeling properties of reseeded hMSCs in a microenvironment that mimics in vivo environment faithfully.

## Methods

### Nanogroove fabrication

UV-assisted capillary molding system was used to fabricate the nanogrooved surfaces (Additional file 1: Figure S1). Silicon masters with areas of 25 × 25 mm^2^ were prepared by standard photolithography and dry etching for replication of nanogroove. Regularly-spaced nanogrooves had 400 nm of width and two different gaps of 400, 800 nm (spacing ratio: 1:1, and 1:2, respectively). To form negative replica, a mixture of perfluoropolyether (PFPE) precursor (Solvey Solexis, MD700, Korea) and 3% of 2-hydroxy-2-methylpropiophenone (Sigma-aldrich, USA) was dropped onto silicon master and dispensed when brought into contact with PET film (Sunkyung Chemical, Korea). PET film was detached after a few seconds of UV treatment. A negative replica on the PFPE film was used to replicate nanogroove onto coverglass (Φ 25 mm, Paul Marienfeld GmbH & Co. KG, Germany). First, cover glassed was modified by glass primer (Minuta Technology Co., Ltd., Korea) and placed in a dry oven preheated to 70 °C for 20 min. Then a UV-curable polyurethane acrylate (PUA) precursor (Minuta Technology Co., Ltd., Korea) was dropped on the nanogrooved cover glass and dispensed when brought into contact with PFPE mold. The cover glasses were treated with UV for 2 min and the PFPE mold was carefully peeled off. The samples were treated with UV overnight to stabilize the nanogroove. The flat surface was also generated on the same coverslip with the same process to maintain the same experimental conditions.

### Cell culture

Human mesenchymal stem cells (purchased from Lonza Group Ltd., Basel, Switzerland) were cultured on tissue flask at initial seeding density of 5×103 cells/cm2 and expanded in the growth medium MSCGMTM Single Quots ™ (Lonza, Switzerland) supplemented with 50 ml mesenchymal stem cell growth supplement (MCGS) (Lonza, Switzerland), 10 ml L − glutamine, and 0.5 ml GA − 1000 (Lonza, Switzerland) at 37^°^C and 5 % CO2. Cells were seeded at passage 5 on the nanogrooved and flat samples with the density 104 cells/cm2 and cultured for 2 weeks for decellularization. Cells re − seeded on the decellularized matrix and their control samples had density 5×10^3^ cells/cm^2^. Media was changed every 3–4 days.

### Decellularization

The cells were washed with DPBS and then removed by incubation with 0.5% Triton X-100 containing 20 mM NH4OH in DPBS for 3 min at room temperature. After gentle washing with DPBS 3 times, the decellularized matrixes were used for further experiments.

### Immunocytochemistry

Cells were stained after 1 and 14 days of culture. First, cells were washed with DPBS and fixed with 4% formaldehyde in PBS for 10 min at room temperature. Then, cells were permeabilized in 1% BSA and 0.3% Triton X-100 in PBS for 10 min. After washing the permeabilizing solution, cells were incubated with the targeted specific primary antibodies overnight at 4 °C: anti-fibronectin (1:200 dilution, Santa Cruz, Deleware, USA), anti-collagen type I (1:200 dilution, Abcam, Cambridge, UK), anti-collagen type II (1:200 dilution, Abcam, Cambridge, UK), anti-collagen IV (1:200 dilution, Abcam, Cambridge, UK) and anti-laminin (1:100 dilution, Sigma, St. Louis, USA). After washing cells 3 times with DPBS, cells were incubated with the secondary antibody (1:300 dilution, Goat anti-rabbit IgG –TRITC) (Sigma, St. Louis, USA) for 1 h at room temperature. Then they were washed again with DPBS 3 times. F-actin was stained with by phalloidin–tetramethylrhodamine B isothiocyanate (1:200, Sigma-Aldrich, USA) for 45 min. Finally, cell nucleus was stained with DAPI (1:1000, Sigma-Aldrich, USA) for 2 min and was washed with DPBS.

### Elongation and alignment characterization

For cell morphology quantification, elongation analysis was done for cells on the nanogrooved and control samples fixed at day 1. At least 8 separate regions of each sample were photographed and at least 100 cells were used for morphology characterization. The images were analysed with ImageJ NIH image processing software (NIH, Bethesda, MD, USA). The elongation (E) parameter indicates the extent of elongation. It was calculated as one minus the ratio of the short axis (S) of minimum bounding rectangle over the long axis (L) of minimum bounding rectangle. Bigger E factor is the cell became elongated, $E=1-\frac{S}{L}$.

Both cell nuclei elongation and cell body elongation were analysed for cells seeded for decellularization. Vinculin length and orientation of the re-seeded cells at day 1 were analysed with ImageJ NIH image processing software (NIH, Bethesda, MD, USA).

### Real-time quantitative reverse transcription PCR (qRT-PCR)

First, mRNAs were extracted from at least 3 samples of each group after 1, 7, and 14 days of culture. Cells were disrupted and total RNAs were extracted with Isol RNA Lysis Reagent (5 prime, Hilden, Deutschland). Then chloroform (Sigma-Aldrich) was added for stabilization of RNAs. After 10 min, samples were centrifuged in 14,000 rpm and supernatant were collected into new tubes for RNA precipitation by adding isopropanol (Sigma-Aldrich) and glycogen (Roche, Basel, Switzerland). Concentrations of precipitated mRNAs were measured by NanoDrop® ND-1000 spectrophotometer (NanoDrop Technologies, Wilmington, DE, USA). cDNA was synthesized by RT-PCR after concentration is adjusted to 1 μg/μl (Table 1) in PCR System (Bio-Rad, Richmond, CA, USA) with PCR Master Mix (Bio-Rad, USA). Then, real-time quantitative PCR was performed by adding forward and reverse primers with SYBR® Green Master Mix Buffer (Toyobo, Japan), and the synthesized cDNA within the temperature setting. In the last step, mRNA expressions of the ECM protein related genes were quantified. Comparative ΔΔCt method was used to determine the level of mRNA expressions, housekeeping gene GAPDH used to normalize the target gene expressions. Target gene primers used for experiments are listed in Table.

**Table 1:** Sequence lists for the primers used in this study

Gene	Forward Primer (5′ → 3′)	Reverse Primer (5′ → 3′)
GAPDH	GTATGACAACAGCCTCAAGAT	AGTCCTTCCACGATACCAAA
FN1	AGCAGACCCAGCTTAGAGTT	GCAGAAGTGTTTGGGTGACT
COL1A1	GGGCCAAGACGAAGACAT	CAACACCCTTGCCGTTGTCG
COL4A1-A2	CTGGTCCAAGAGGATTTCCA	TCATTGCCTTGCACGTAGAG
COL2A1	GGCAATAGCAGGTTCACGTACA	CGATAACAGTCTTGCCCCCACTT
LAMB1	AACGTGGTTGGAAGAACCTG	ACACTCCCTGGAAACAGTGG
DCN	AATTGAAAATGGGGCTTTCC	GCCATTGTCAACAGCAGAGA
BGN	GGACTCTGTCACACCCACCT	AGCTCGGAGATGTCGTTGTT
ACAN	TGCGGGTCAACAGTGCCTATC	CACGATGCCTTTCACCACGAC

### Western blot

Proteins were extracted from cell cultures with RIPA buffer (Sigma-Aldrich) contained Protein Inhibitor Cocktail (Thermo Fisher Scientific, USA). For protein concentration measurement, Bradford assay was done with absorbance measurement at 595 nm wavelength. Protein aliquots containing same amount of total protein were loaded on 8% sodium dodecyl sulfate-polyacrylamide gel electrophoresis (SDS-PAGE) gels. Then separated proteins were transferred to the nitrocellulose membrane for the ECM protein detection. The membranes were incubated with primary antibodies overnight at 4 °C. After washing membranes with TBST (Tris buffered saline and Tween 20) solution, horseradish peroxiodase (HRP) conjugated secondary antibodies for 1 h at room temperature. Membranes were washed with TBST again then, HRP conjugated antibodies reacted with combined stable peroxide solution and luminol solution (Thermo Fisher Scientific, USA) where chemical reaction emitted light at 425 nm. CCD camera and phosphorimagers using chemiluminescence image analyser (ImageQuant LAS 4000 mini, GE healthcare, Little Chalfont, USA) captured emitted light.

### Statistical analysis

Data are presented as the mean ± standard error of the mean (SEM). Student’s t-test was used to determine levels of significance for comparisons between two independent samples. Groups were compared by one-way analysis of variance (ANOVA) with Tukey’s post-hoc test was applied to significant main effects. Differences were considered statistically significant when *p*-value is *p* < 0.05 (*), *p* < 0.01 (**), or *p* < 0.001 (***).

## Results

### hMSC morphology

**Fig. 1:**
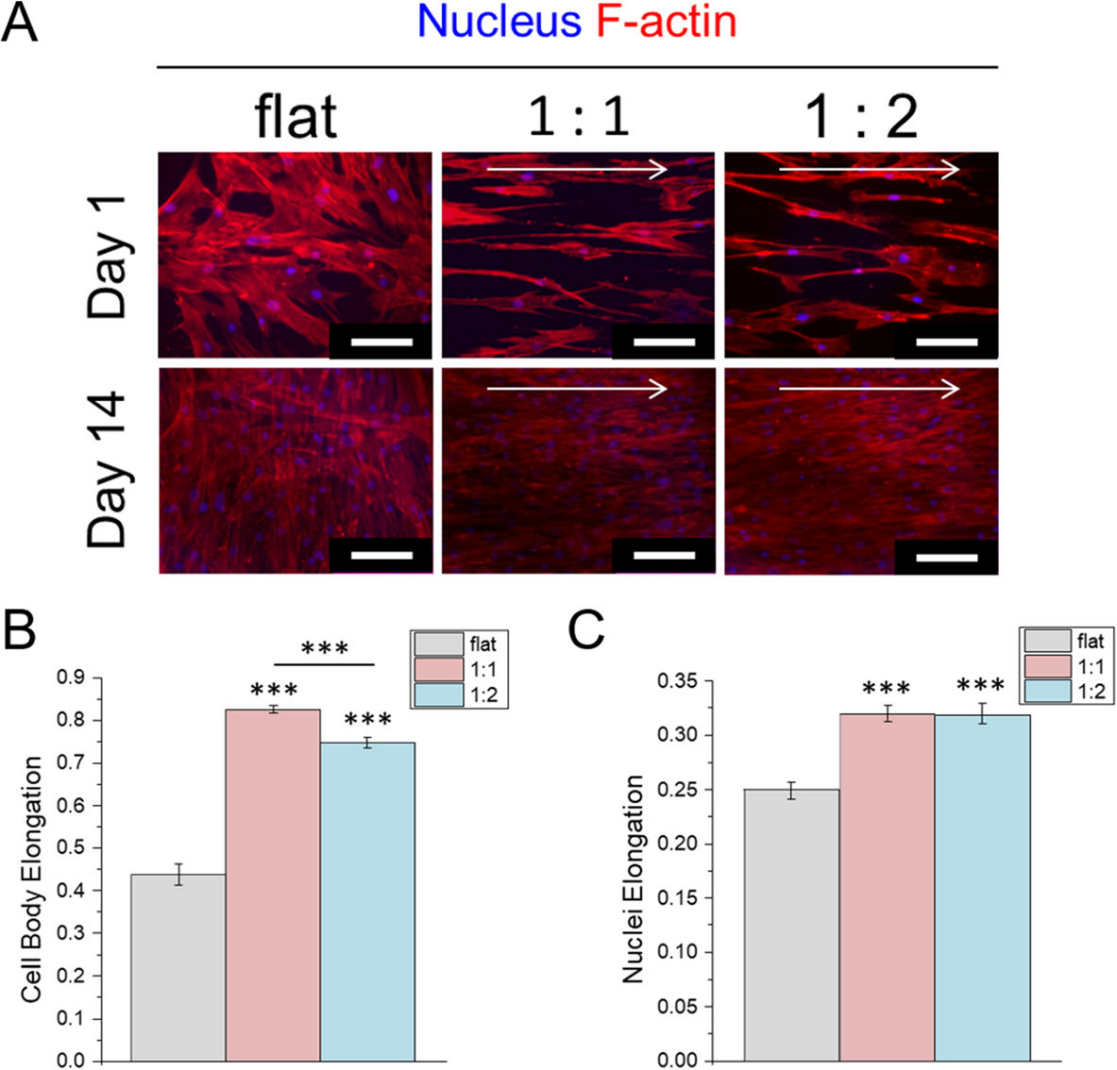
Morphology and elongation characterization of MSCs on nanopattern. **a** F-actin staining of MSC on Day:1 and Day:14. (Scale bar: 150 μm). **b** Cell body elongation. **c** Nuclei Elongation. Statistical significance to flat control (***: *p* < 0.001)

hMSCs were seeded on a flat surface and nanogrooved surfaces and stained after 1 and 14 days for analysis of their morphology and orientation. The nanogrooves comprises 400-nm-wide nanogrooves separated by 400 or 800 nm (spacing ratios of 1:1 and 1:2, respectively). The hMSCs cultured on the nanogrooved surface were highly aligned and elongated in the direction of the nanogrooves; in contrast, hMSCs cultured on the flat control surface exhibited a random orientation (Additional file 1: Figure S2, Fig. 1a). The cytoskeleton and nuclei were significantly more elongated parallel to the nanogrooves than were those of cells on the flat control surface (Fig. 1a-c). The magnitude of cytoskeletal elongation of hMSCs cultured on the nanogrooved surface at a 1:1 spacing ratio was significantly higher than that of hMSCs cultured on the nanogrooved surface with a 1:2 spacing ratio (Fig. 1b).

### ECM protein production

**Fig. 2:**
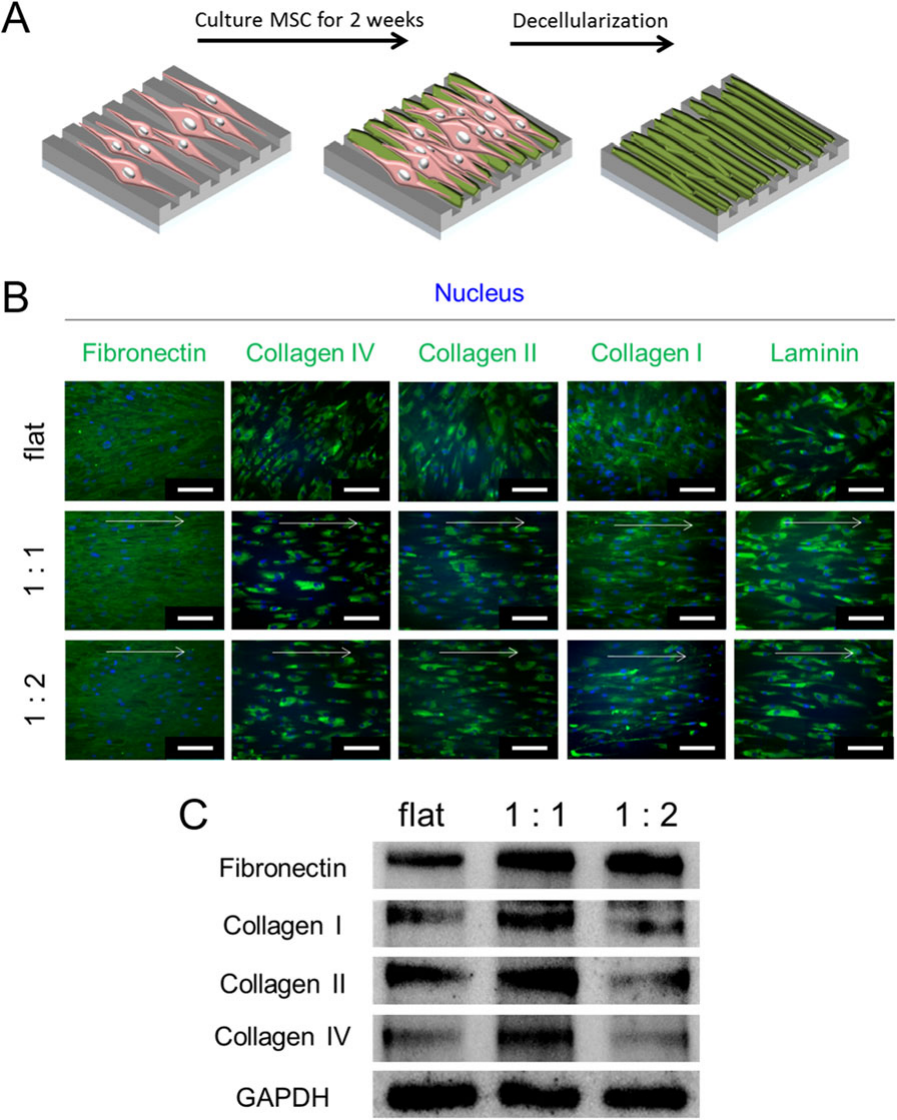
Analysis of the ECM protein composition and orientation on nanopattern. **a** Schematic illustration for MSC culture on nanopattern for decellularization. **b** Immunofluorescence staining of ECM proteins Fibronectin, Collagen I, Collagen II, Collagen IV, and Laminin at Day14 before decellularization. Arrow indicates nanogroove direction, scale bar: 150 μm. **c** Western Blot for relative concentration of ECM proteins Fibronectin, Collagen I, Collagen II, Collagen IV at Day14

To investigate the effect of nanogrooves on hMSC-derived ECM structure and composition, hMSCs were cultured for 2 weeks to full confluency and immunostained for the following ECM proteins: fibronectin; collagen types I, II, and IV; and laminin (Fig. 2a, b). Immunofluorescence staining showed that hMSCs on both the flat and nanogrooved surfaces produced abundant fibronectin. In addition, collagen types I, II, and IV, and laminin, were detected on both the flat and nanogrooved surfaces. The architectures of the ECM on the nanogrooved surface were highly aligned in the direction of the nanogrooves; in contrast, hMSCs on the flat surface exhibited a random orientation. To quantity ECM protein production by the hMSCs, western blot analysis was performed (Fig. 2c). hMSCs cultured on the nanogrooved surface produced more fibronectin than did those on the flat surface. Even though production of fibronectin by the hMSCs on the nanogrooved surfaces were similar to the others, production of collagen types I, II, and IV by hMSCs on the nanogrooved surface with a 1:1 spacing ratio was higher than that of hMSCs on the nanogrooved surface with a 1:2 spacing ratio, and on the flat surface.

### Decellularized-MSC ECM

**Fig. 3:**
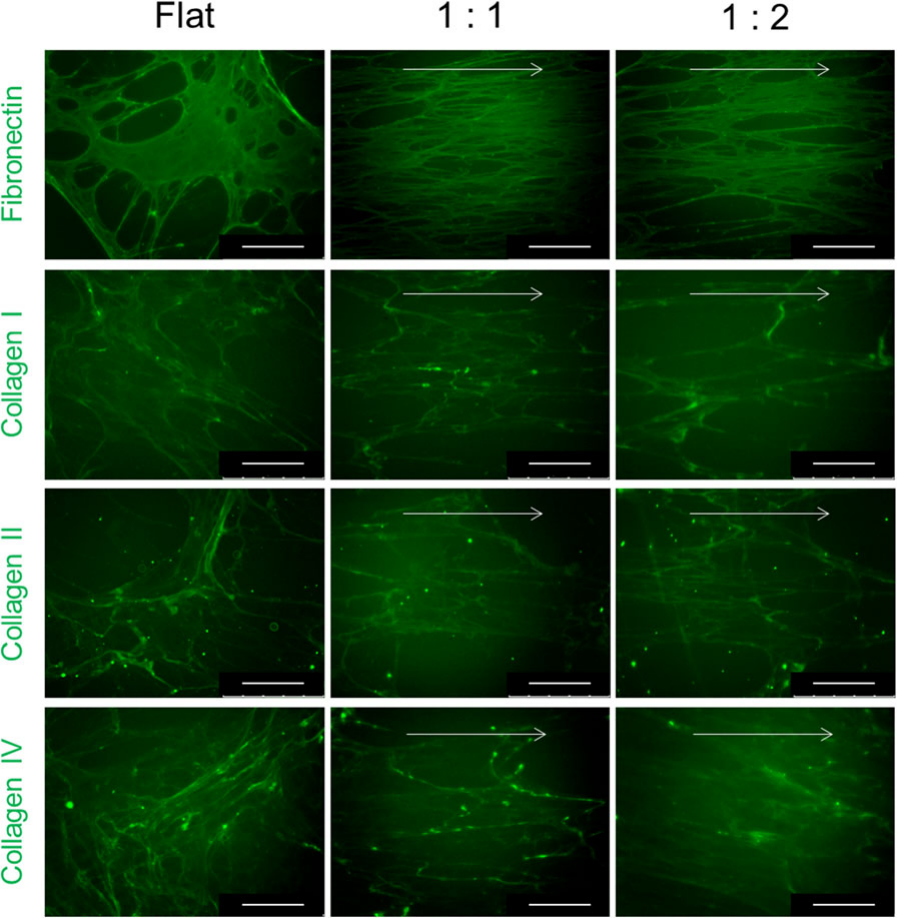
Immunofluorescence staining of the decellularized-MSC ECM, Scale bar: 150 μm

To investigate ECM structure and composition after decellularization, the decellularized matrices were stained for major ECM proteins (Fig. 3). Staining results showed that the ECM composition and structure produced by hMSCs on the flat and nanogrooved surfaces were well-maintained after decellularization. Although the structure of ECM does not show significant difference between nanogrooved surface after the decellularization, production of collagen types I, II, and IV by hMSCs on the nanogrooved surface with a 1:1 spacing ratio was higher than that of hMSCs on the nanogrooved surface with a 1:2 spacing ratio before the decellularization (Fig. 2c). Therefore, the decellularized ECM on the nanogrooved surface with a 1:1 spacing ratio was used in subsequent experiments.

### Characterization of re-seeded hMSCs

**Fig. 4:**
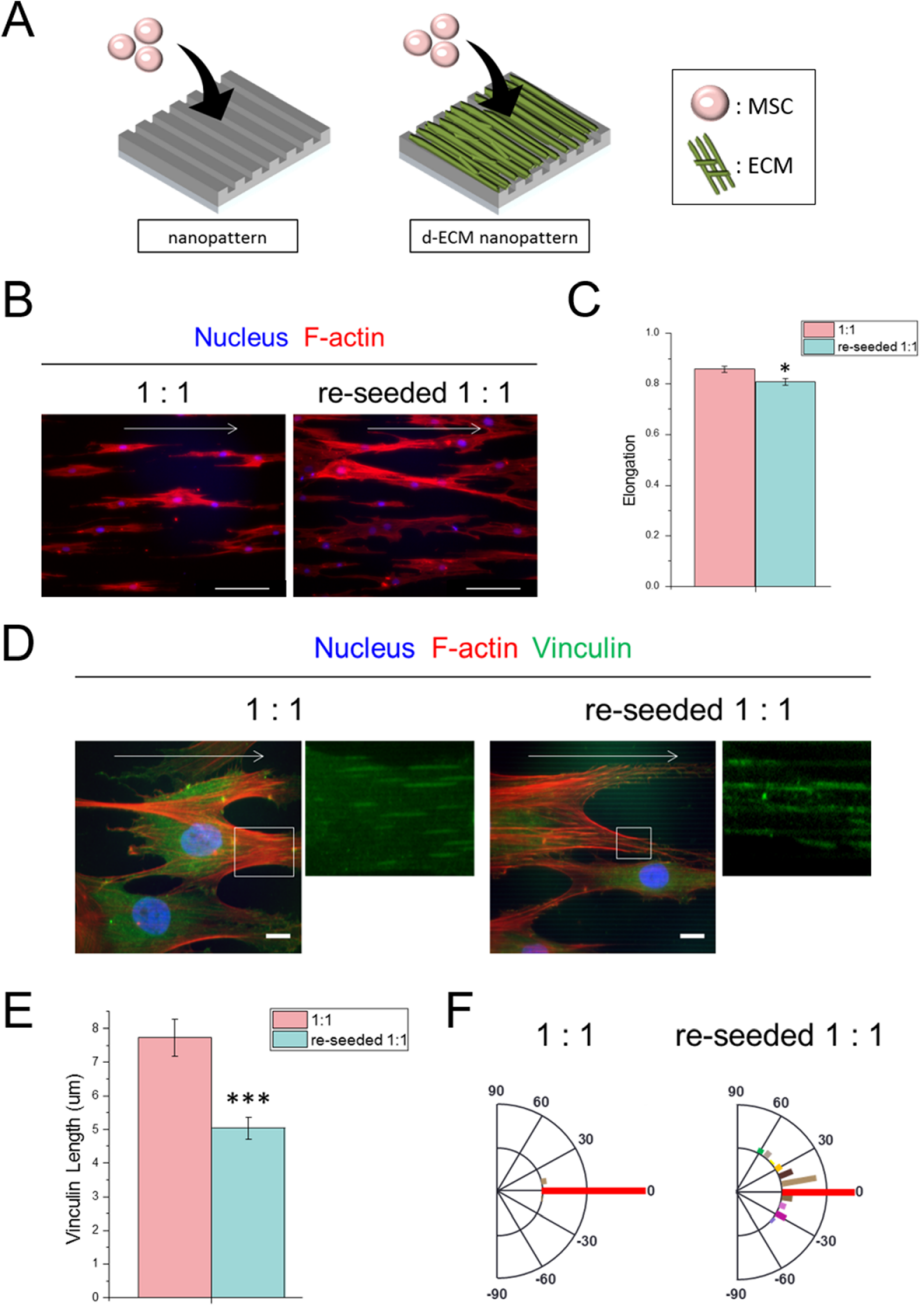
Morphology and elongation characterization of the re-seeded MSCs. **a** Schematic illustration of re-seeding MSC on decellularized-MSC ECM at Day1. **b** Immunofluorescence staining of f-actin, arrow indicates nanogroove direction, scale bar: 150 μm at Day1. **c** Elongation of the re-seeded MSCs on the decellularized ECM where *** = significant to 1:1 control (p < 0.001). **d** Immunofluorescence staining of focal adhesion, arrow indicates nanogroove direction, scale bar: 15 μm. **e** Vinculin length of re-seeded MSCs where * = significant to 1:1 control (*p* < 0.05). **f** Polarization graph of focal adhesions on nanopattern

Next, we examined the effect of an hMSC-derived ECM sheet on the expression of ECM proteins by hMSCs, particularly the influence of the nanoscale architecture of the ECM sheet. A nanogrooved surface (1:1 spacing ratio) without an hMSC-derived ECM was used as a control (Fig. 4a). After 1 day of culture, the re-seeded hMSCs were stained for morphological analysis (Fig. 4b). The hMSCs re-seeded on the hMSC-derived ECM sheet showed a less elongated morphology than those on the bare nanogrooved surface (Fig. 4c).

Vinculin staining was performed to further characterize hMSC morphology (Fig. 4d) by analyzing the formation of focal adhesions by cells on the decellularized ECM. The vinculin length of hMSCs re-seeded on the nanostructured ECM sheet (with ECM sheet) was significantly shorter (Fig. 4e) than that of hMSCs re-seeded on the bare nanogrooved surface. In addition, the distribution of focal adhesions in the re-seeded hMSCs on the ECM sheet was less aligned than that of the control hMSCs (Fig. 4f). Therefore, the nano-engineered decellularized ECM influenced the phenotypic features of re-seeded hMSCs.

**Fig. 5:**
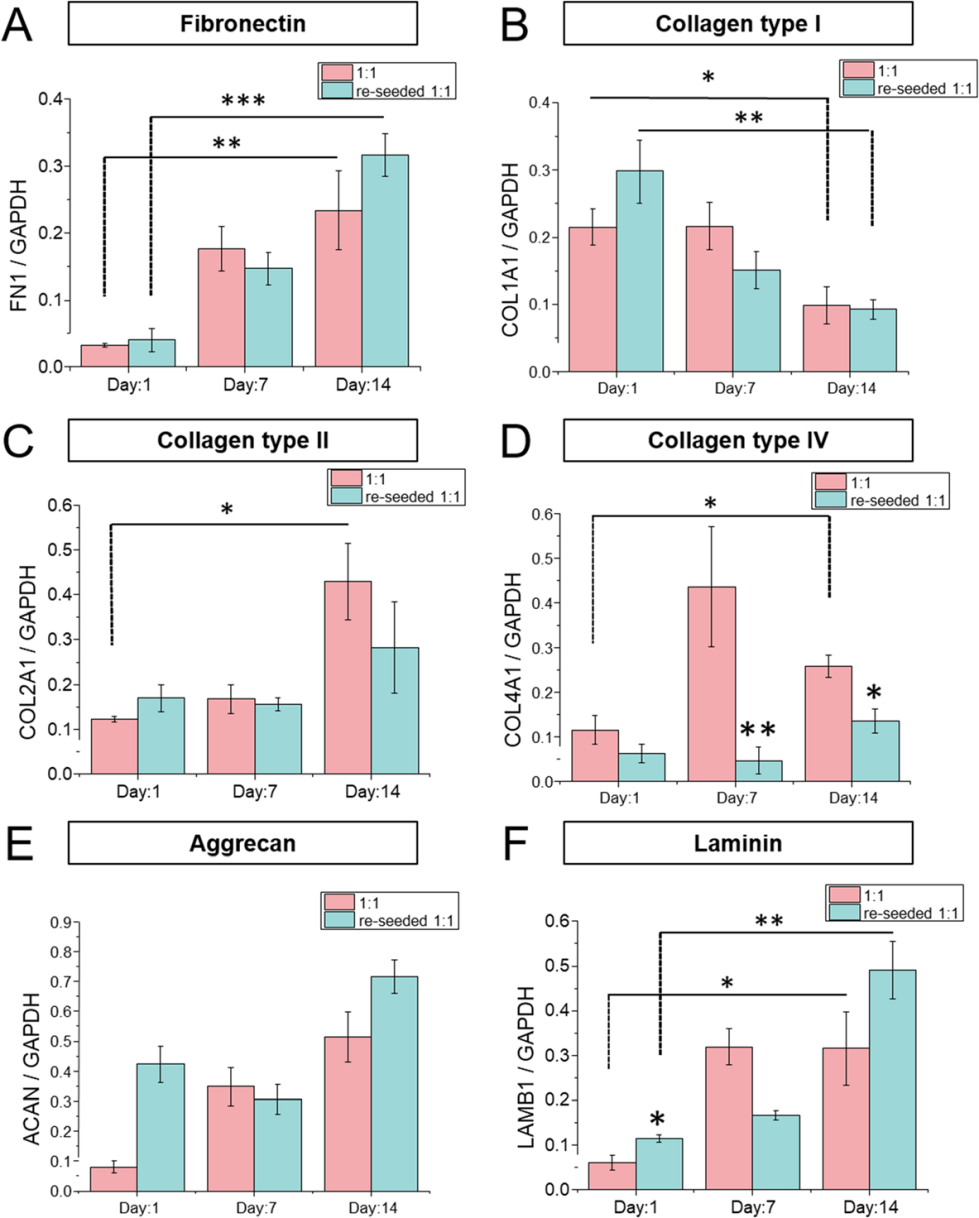
ECM protein production of the re-seeded MSC. Expression levels of ECM protein-related genes FN1 (fibronectin), COL1A1 (collagen I), COL2A1 (collagen II), COL4A1 (collagen IV), ACAN (aggrecan), LAMB1 (laminin) relative to expression level of GAPDH. Pink bar: 1:1 control, Mint bar: re-seed 1:1. Statistical significance: *p< 0.05, **p< 0.01, and ***p< 0.001 to 1:1control

Next, quantitative reverse transcription-polymerase chain reaction qRT-PCR (analysis) was performed to quantify the expression of genes encoding ECM proteins by hMSCs re-seeded on nanogrooved surfaces with and without a decellularized ECM. *FN1* and *COL2A1* expression by the re-seeded hMSCs increased continually and was not significantly different from the controls (Fig. 5a, c). *COL1A1* expression by the re-seeded hMSCs was lower than that of the controls (Fig. 5b), while *LAMB1* expression by the re-seeded hMSCs was higher than that of the controls at 1 day and increased further at 2 weeks (Fig. 5f). *COL4A1* expression was similar in both groups and significantly decreased after 2 weeks (Fig. 5d). *ACAN* expression by the re-seeded hMSCs was higher than that of the controls at day 1 and increased further at 2 weeks (Fig. 5e). Therefore, the nano-engineered decellularized ECM contributed to modulation of ECM production by hMSCs.

## Discussion

Nanotopography as a biophysical microenvironment controls stem cell fate [15]. Together with directional and structural stimuli, ECM molecules regulate cell phenotype. Within such microenvironments, hMSCs can be influenced by ECM-derived cytoskeletal reorganization, assembly of focal adhesions, and conformational adaptation of chromatin structure; these processes ultimately alter cell function [18, 32–35].

In this study, we examined the effect of nanotopography and an hMSC-derived ECM on the expression of ECM proteins to investigate ECM-mediated adaptation of hMSCs to their microenvironment. As reported previously [18, 32, 34–36], the hMSCs were aligned in the direction of the nanogrooves (Fig. 1a), indicating that geometrical cues from nanogrooves influenced cytoskeletal structure and the orientation of focal adhesions. The geometrical cues provided by nanotopography have a considerable effect on the adhesion, migration, proliferation, and differentiation of hMSCs [18, 32–35]; thus, changes in the morphological phenotype of hMSCs lead to conformational adaptations of chromatin structure and organization, which ultimately influence cell function [18, 33]. We hypothesized that geometrical-cue-induced morphological changes affect ECM production by the hMSCs. Cells cultured on the nanogrooved surface at a 1:1 spacing ratio had the most elongated morphology (Fig. 1b) and higher amount of ECM protein (Fig. 2c). Our results showed that the composition and structure of ECM proteins were influenced by the nanogrooves, and the highest-density nanopattern exerted the greatest effect. This indicated that nanopattern-derived geometrical cues impact on ECM production by hMSCs.

The composition of hMSC-derived ECM is dependent on the culture conditions [28–30], and can facilitate maintenance of stemness or enhance differentiation into specific cell lineages [16, 17, 24–31]. Our findings show that a nano-engineered hMSC-derived matrix sheet altered ECM production by re-seeded hMSCs. hMSCs re-seeded on a fibronectin-rich decellularized ECM sheet had a distinct phenotype, characterized by shorter vinculin molecules (Fig. 4e) with a random distribution compared to cells cultured in the absence of an ECM (Fig. 4f). This reduction in vinculin length could indicate cell elongation, which is involved in phenotypic alterations. Thus, the presence of a decellularized ECM on the dense nanogrooved surface reduced the sensitivity of the re-seeded hMSCs to the effect of nanotopography and resulted in randomly distributed focal adhesions.

Gene expression analysis revealed that the nano-engineered hMSC-derived ECM sheet influences the ECM composition of re-seeded hMSCs. Fibronectin, which affects cell adhesion, migration, and growth, was secreted in large quantities by the hMSCs [4, 37]. Production of fibronectin by hMSCs is reportedly stable under different culture conditions and differentiation states [28–30]. As expected, *FN1* expression by the re-seeded hMSCs was not significantly different from that of the controls and increased continually. Expression of *COL4A1* and *LAMB1*—which encode the basal lamina proteins, laminin and collagen IV—by re-seeded hMSCs was down- and up-regulated, respectively, compared to the controls. The presence of collagen IV in the decellularized ECM may contribute to regulation of ECM production in hMSC, causing decrease in associated gene expression. In contrast, because laminin is sensitive to the decellularization solution and easily washed away, the absence of laminin in the decellularized ECM might explain the increased *LAMB1* expression by the re-seeded hMSCs.

The expression of *COL1A1*, *COL2A1*, and *ACAN*, which encode collagen types I and II, and aggrecan, respectively, showed an interesting pattern of changes. Collagen type I is related to osteogenesis of hMSCs and *COL1A1* expression increases during differentiation of hMSCs into osteoblasts [28]. In contrast, collagen type II and aggrecan are related to chondrogenesis of hMSCs and their expression is upregulated during differentiation of hMSCs into chondrocytes [28, 30]. *COL1A1* expression by the re-seeded hMSCs was downregulated, and that of *COL2A1* and *ACAN* upregulated, at 2 weeks, suggesting that the microenvironment is conducive to chondrogenesis. This likely involved upregulation of *COL2A1* and *ACAN* and downregulation of *COL1A1*. Although the decellularized-ECM on the nanogrooved surface did not induce differentiation in the absence of differentiation-inducing medium, the nano-engineered hMSC-derived matrix modulated the expression of ECM proteins by hMSCs.

## Conclusions

We report here the effect of a nano-engineered hMSC-derived ECM on a nanogrooved surface on hMSC phenotype and ECM production. Due to their elongated morphology and chromatin structure adaptation, hMSCs on the dense nanogrooved surface produced higher levels of ECM proteins and a re-organized ECM. The decellularized ECM with an aligned nanostructure provided biochemical and geometrical cues that altered the focal adhesion polarization of re-seeded hMSCs. In addition, the expression of chondrogenesis-related genes was upregulated at 2 weeks, suggesting that the nano-engineered hMSC-derived ECM induced a microenvironment conducive to chondrogenesis. Therefore, nanotopography influences ECM production by hMSCs. Our results suggest that decellularized ECM on nanostructures cause hMSCs to remodel their ECM microenvironment to guide differentiation into a specific cell lineage.

## Additional file

Additional file 1: **Figure S1.** Schematic of nanopattern fabrication used in this study. **Figure S2.** Polarization graph of focal adhesions of the cells on flat surface. **Figure S3.** Immunofluorescence staining of the fibronectin and nucleus of hMSCs before and after decellularization, Scale bar: 150 μm. (DOCX 490 kb)
