# Supplementary material for: Nanoengineered, cell-derived extracellular matrix influences ECM-related gene expression of mesenchymal stem cells
Source: Biomater Res. 2018 Oct 5;22:32. doi: 10.1186/s40824-018-0141-y (PMC6173882; doi:10.1186/s40824-018-0141-y)
Supplement: Supplementary file 1 — Figure S1. Schematic of nanopattern fabrication used in this study. Figure S2. Polarization graph of focal adhesions of the cells on flat surface. Figure S3. Immunofluorescence staining of the fibronectin and nucleus of hMSCs before and after decellularization, Scale bar: 150 μm. (DOCX 490 kb) [file 40824_2018_141_MOESM1_ESM.docx]

**Additional file 1**

**Nanoengineered, Cell-derived Extracellular Matrix Influences ECM-related Gene Expression of Mesenchymal Stem Cells**

Hatice O. Ozguldez, Junghwa Cha, Yoonmi Hong, Ilkyoo Koh, Pilnam Kim*


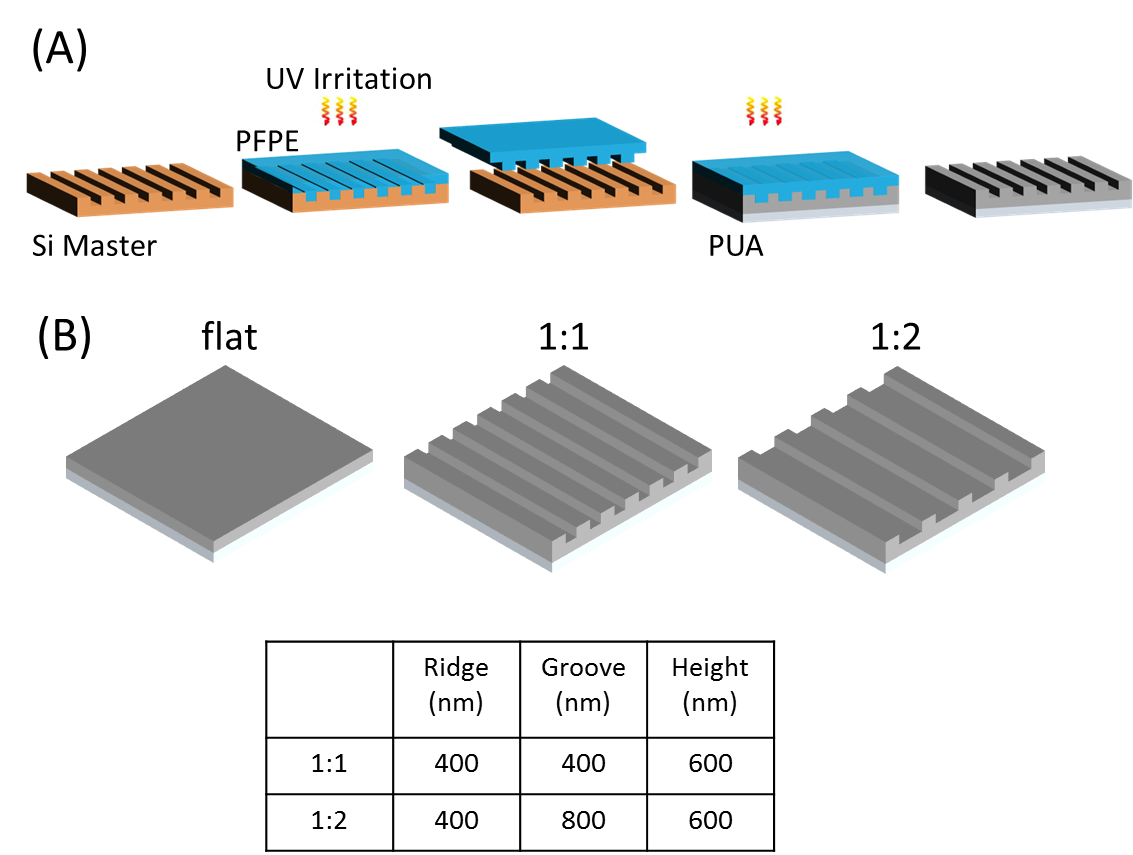


**Figure S1.** Schematic of nanopattern fabrication used in this study.


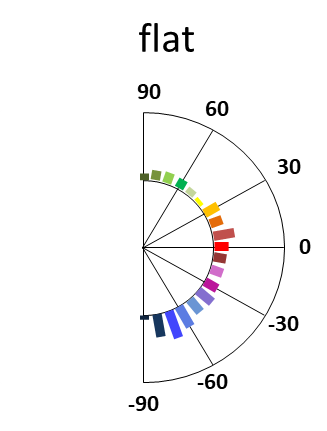


**Figure S2.** Polarization graph of focal adhesions of the cells on flat surface.


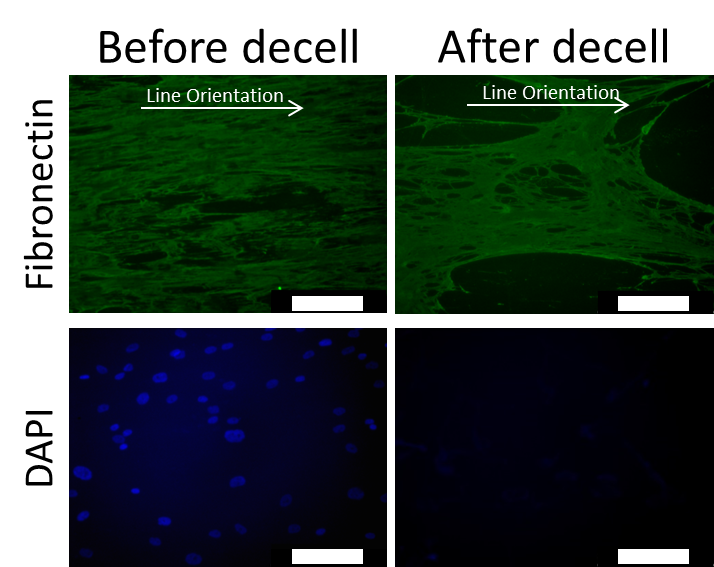


**Figure S3.** Immunofluorescence staining of the fibronectin and nucleus of hMSCs before and after decellularization, Scale bar: 150 μm
